# Malignant Peripheral Nerve Sheath Tumors State of the Science: Leveraging Clinical and Biological Insights into Effective Therapies

**DOI:** 10.1155/2017/7429697

**Published:** 2017-05-16

**Authors:** AeRang Kim, Douglas R. Stewart, Karlyne M. Reilly, David Viskochil, Markku M. Miettinen, Brigitte C. Widemann

**Affiliations:** ^1^Center for Cancer and Blood Disorders, Children's National Health System, 111 Michigan Ave NW, Washington, DC 20010, USA; ^2^Clinical Genetics Branch, Division of Cancer Epidemiology and Genetics, National Cancer Institute, 9609 Medical Center Drive, Room 6E450, Bethesda, MD 20892, USA; ^3^Rare Tumors Initiative, OD, CCR, National Cancer Institute, 37 Convent Drive, Bethesda, MD 20814, USA; ^4^University of Utah, 295 Chipeta Way, Salt Lake City, UT 84108, USA; ^5^Center for Cancer Research, National Cancer Institute, 10 Center Drive, Room 2S235C, Building 10, Bethesda, MD 20892, USA; ^6^National Cancer Institute, Pediatric Oncology Branch, 10 Center Drive, Room 1-3742, Building 10, Bethesda, MD 20892, USA

## Abstract

Malignant peripheral nerve sheath tumor (MPNST) is the leading cause of mortality in patients with neurofibromatosis type 1. In 2002, an MPNST consensus statement reviewed the current knowledge and provided guidance for the diagnosis and management of MPNST. Although the improvement in clinical outcome has not changed, substantial progress has been made in understanding the natural history and biology of MPNST through imaging and genomic advances since 2002. Genetically engineered mouse models that develop MPNST spontaneously have greatly facilitated preclinical evaluation of novel drugs for translation into clinical trials led by consortia efforts. Continued work in identifying alterations that contribute to the transformation, progression, and metastasis of MPNST coupled with longitudinal follow-up, biobanking, and data sharing is needed to develop prognostic biomarkers and effective prevention and therapeutic strategies for MPNST.

## 1. Introduction

Neurofibromatosis type 1 (NF1) is an autosomal dominant, pan-ethnic disorder with an incidence of 1 : 3000 [1]. NF1 is characterized by diverse, progressive cutaneous, neurologic, skeletal, and neoplastic manifestations with limited therapeutic options. The leading cause of death in NF1 patients is the malignant peripheral nerve sheath tumor (MPNST), a highly aggressive soft tissue sarcoma [2]. Half of all MPNST develop in individuals with NF1, with a 5-year survival of about 20% to 50%, and the outcome is especially dismal in those with unresectable or metastatic disease [2, 3]. Most (65–88%) NF1 MPNST arise from plexiform neurofibromas (PN) [4], benign peripheral nerve sheath tumors that are a hallmark of NF1. The only known definitive therapy for MPNST is surgical resection with wide negative margins [4, 5], which is often not feasible or indicated due to location, size, and metastases [6, 7].

A 2002 MPNST consensus statement reviewed current knowledge, provided guidance for the diagnosis and management of MPNST, and identified research priorities [8]. While little progress has been made in the development of more effective therapies since then, there have been substantial advances in understanding MPNST natural history, biology, and preclinical modeling, and preclinical and clinical trial consortia have been established (Table 1). In this review, we update progress since 2002 in the (1) natural history of peripheral nerve sheath tumors, (2) pathogenesis of MPNST, (3) development of preclinical models, and (4) management and clinical trials for MPNST.

## 2. Natural History of Peripheral Nerve Sheath Tumors

**Figure 1:**
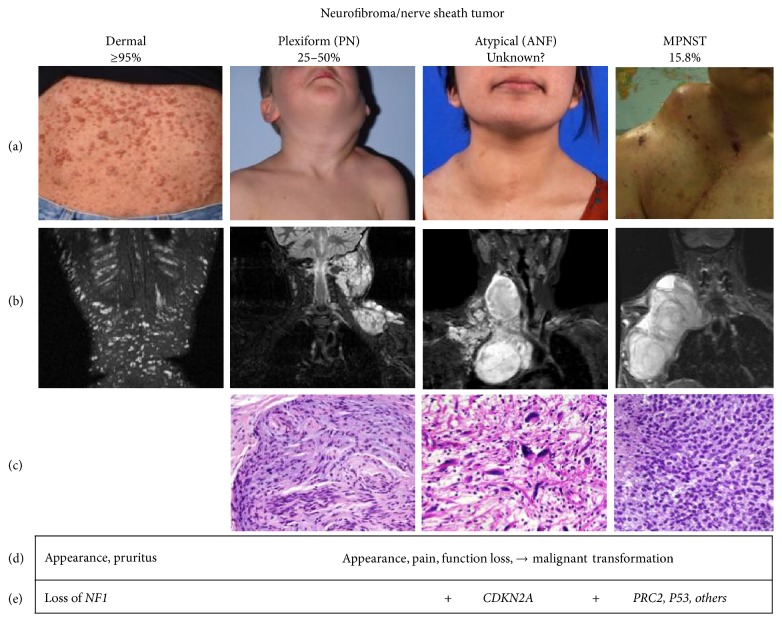
Pathogenesis of peripheral nerve sheath tumors in NF1. Percentages below each tumor type is the range of lifetime prevalence in individuals with NF1. Representative clinical photograph (a), MRI imaging (b), histology (c), clinical symptomology (d), and genetic features (e) of each tumor type are given. Histologically, plexiform neurofibroma shows mixture of areas of hypercellularity in the absence of other atypical features. Atypical neurofibroma shows atypical nuclei and higher cellularity. In contrast, MPNST are highly cellular with high mitotic activity and areas of necrosis.

PN, a cardinal feature of NF1, are identified in up to 50% of individuals with NF1 [9]. They are a major source of morbidity [10], causing disfigurement, impairment of nerve function, pain, and in some cases transform to MPNST (Figure 1) [2, 3]. Magnetic resonance imaging (MRI) and fluorodeoxyglucose- (FDG-) positron emission tomography (PET) are utilized in the diagnosis of malignant transformation with features to aid in distinguishing MPNST from PN [11–14]. Since 2002, the use of whole-body and targeted longitudinal MRI with volumetric analysis has permitted the sensitive and reproducible characterization of PN growth [15–19]. Most PN growth occurs in children, and substantial PN volume increase is infrequent in adults. This is in contrast to distinct nodular lesions (DNL) which have been identified using longitudinal whole-body MRI and display different imaging and growth characteristics [20, 21]. On Short T1 Inversion Recovery (STIR) MRI, these lesions are nodular, ≥3 cm in longest diameter, and well demarcated and lack the “central dot” sign characteristic of PN. MRI imaging for MPNST demonstrate irregularly shaped, ill-defined margins, intratumoral lobulation, and inhomogeneous contrast enhancement [12]. DNL emerge after early childhood, their growth rate is not age-related, and they are frequently higher than that of surrounding or adjacent PN. In contrast to typical PN, most DNL are FDG-avid on FDG-PET [21, 22] more like MPNST [13]. Biopsy and excision of some radiographically detected DNL reveal histologically atypical neurofibromas (ANF). ANF share some features of low-grade MPNST and recognition of transformation of ANF to MPNST suggests that ANF are premalignant lesions of MPNST rather than variants of PN [23]. ANF have increased variable cellularity and have cells with enlarged, hyperchromatic nuclei and more pronounced fascicular growth [23, 24]. Taken together, these findings suggest that DNL have a distinct underlying biology compared to PN [20]. Genomic findings of* CDKN2A/B* loss in ANF and MPNST (but not PN) further support the hypothesis that ANF are precursor lesions for MPNST [22, 23, 25]. In a retrospective analysis of 76 ANF diagnosed in 63 patients with NF1, the majority (*n* = 57) were resected and have not recurred [22]. However, four ANF transformed into high grade MPNST. Sixteen patients had a history or developed MPNST in a different location, and patients with ANF may be at greater risk of developing MPNST [22]. Limited correlation of clinical outcome in surgical excision of ANF suggests that these lesions may not require aggressive surgery as MPNST. In a retrospective review of 23 patients who underwent surgical resection of a plexiform neurofibroma pathologically diagnosed as either low-grade MPNST or ANF had disease-specific survival of 100% with a median follow-up of 47 months despite 78% (18/23) of patients having microscopically positive margins [26]. No patients developed pulmonary metastasis. Further study is warranted, but focal surgical resection of premalignant ANF may play an important role in the prevention of MPNST.

## 3. Pathology of MPNST

Sarcoma arising from the peripheral nerve sheath is readily diagnosed as MPNST if the tumor clearly has nerve elements or arises in the context of NF1. Otherwise, the diagnosis of MPNST is more difficult, with a broad differential diagnosis of other sarcomas, and requires an extensive clinicopathologic assessment of immunohistochemical (IHC) markers, tissue ultrastructure, and histologic findings [24, 27] to firmly establish a tumor diagnosis. High-grade MPNST are highly cellular with many mitotic figures and areas of necrosis. Low-grade MPNST are less cellular, have few mitotic figures and no areas of necrosis, and are difficult to distinguish from benign cellular neurofibromas and ANF. Various histologic patterns can coexist within a single specimen, making it imperative to examine as much of the tumor as possible to arrive at an appropriate diagnosis and grade [28]. Small biopsies are usually inadequate for clinical decision-making due to this intratumor heterogeneity.

IHC studies are helpful in distinguishing high-grade MPNST from other sarcomas but are less helpful in distinguishing ANF from low-grade MPNST. Typical staining includes in situ antibody studies on multiple formalin-fixed sections for S100 (calcium-binding motif as Schwann cell marker), Ki-67 (nuclear nonhistone protein marker of cell proliferation), TP53 (tumor suppressor marker for transformation), CD34 (sialomucin glycoprotein as nonspecific marker of endothelium and hematopoietic stem cells), and p16INK4a (cell-cycle inhibitory protein marker that is inactivated in MPNST). A standardized set of IHC markers has not been routinely applied to peripheral nerve sheath tumors across clinical pathology laboratories. Although they may be useful in characterizing MPNST [29], the pattern of IHC staining has not led to stratification of patients for personalized management of their tumor. Use of genetic markers in these tumors is emerging as another modality to more fully characterize peripheral nerve sheath tumors for clinical intervention.

## 4. Genetics and Genomics of MPNST

MPNST cells harbor complex rearranged genomes. Accumulated evidence suggests that* NF1* loss is necessary but not sufficient for MPNST development. As NF1-associated MPNST progress from* NF1*-nullizygous PN, they acquire mutations in other driver genes (e.g.* TP53* and* CDKN2A*).* NF1* loss is seen in a majority of sporadic MPNST, suggesting that* NF1* is an important tumor suppressor in all MPNST. Genetic alterations of* CDKN2A *and* TP53* are also observed in sporadic and radiation-associated MPNST [30]. Deletion of* CDKN2A* disrupts two encoded proteins (p16INK4A and p19ARF) and their associated regulatory cascades.* CDKN2A* deletions are also observed in ANF [23]. The first study of NF1-associated tumor progression in a single patient from PN to primary MPNST and MPNST metastasis using whole exome sequencing (WES) of biopsies [31] found biallelic* NF1* mutations in all tumor stages, chromosome 17p* (TP53)* loss in primary MPNST and metastasis, and no* CDKN2A* deletions or* EGFR* amplifications. Subsequent cytogenetic and array comparative genomic hybridization (aCGH) studies on MPNST have identified frequent losses on chromosomes 1p, 9p, 11, 12p, 14q, 18, 22q, X, and Y, with focal gains on chromosomes 7, 8q, and 15q [32]. There are no pathognomonic chromosomal translocations in MPNST. Amplification of genes encoding the epidermal growth factor (EGF) receptor, neuregulin-1 (NRG1) coreceptor erbB2, c-Kit, platelet-derived growth factor-*α*, and c-Met has been reported in MPNST [33].

In 2014, somatic mutations in* SUZ12* and* EED* encoding components of the polycomb repressive complex 2 (PRC2) were reported in NF1-associated and sporadic MPNST [30, 34, 35]. PRC2 is a histone methyltransferase and plays a critical role in marking chromatin for silencing. This finding suggests that transformation to MPNST involves a previously unsuspected epigenetic switch and points to potential epigenetic-based therapeutic strategies. Comprehensive genomic characterization of sporadic, NF1-, and radiation-associated MPNST shows recurrent inactivation of PRC2 from somatic mutation of* EED* and* SUZ12* [30, 35]. The* SUZ12* gene encodes a chromatin modifying protein, and its loss enhances colony growth of* NF1*-deficient (but not* NF1* wild-type) glioblastoma cells, suggesting that reduced PRC2 levels might promote tumorigenesis. Furthermore,* SUZ12* ablation causes loss of trimethylation at lysine 27 of histone H3 (H3K27me3) and increased H3K27 acetylation, establishing transcriptional activation marks to recruit bromodomain proteins that are potential drug targets for MPNST [34].

Frequent somatic alterations of* CDKN2A* and* NF1* significantly co-occur with PRC2 alteration.* SUZ12* is located near* NF1* in 17q11.2 and is involved in both type 1 and type 2 microdeletions at the* NF1* locus. Such microdeletions are associated with an increased risk of MPNST [36], leading to a model in which a “third hit” in* SUZ12* (the first two hits being the loss of* NF1* and one copy of* SUZ12* from a 17q11.2 microdeletion) drives transformation to MPNST [30]. PRC2 catalyzes trimethylation of H3K27 and multiple studies have found that significant loss of H3K27me3 in MPNST is associated with poor survival; furthermore, such loss is not observed in PN or ANF [37–39]. H3K27me3 loss or PRC2 mutation may be a useful biomarker to diagnose MPNST [35].

## 5. Preclinical Models

The primary model systems used to study MPNST have been (1) cell lines derived from MPNST patients, (2) xenograft models of patient-derived MPNST cells injected subcutaneously or into the sciatic nerve of immune compromised mice, (3) patient-derived xenografts (PDX) that have not been cultured, and (4) genetically engineered mouse models of sporadic MPNST.

### 5.1. Cell Culture Models

MPNST tumor lines from human and mouse have been used to elucidate the mechanism of action of neurofibromin [40]; study the role of tyrosine kinase receptors [41–47], growth factors [48–50], p53 [51, 52], microRNAs [30, 53], and sex hormones [54–56] in MPNST biology; and examine the effects of chemotherapy [57–67] and viral therapy [68–71] as potential treatments for MPNST. The most commonly used strains for grafting have been S462, ST88-14, and STS26T. STS26T was isolated from a metastatic lesion and has been shown to form metastases when injected into the tail vein of the mouse [62].

At least 33 NF1 or sporadic MPNST lines from primary or metastatic human tumors and mice tumors have been described in the literature to varying degrees (Supplemental Table 1, in Supplementary Material available online at https://doi.org/10.1155/2017/7429697). Mouse tumor lines have been made by isolating tumors with MPNST histology from* Nf1*^−/+^*:Trp53*^−/+^*cis* mice (see below).

### 5.2. Xenograft/Orthograft Models

Over half of the described MPNST tumor lines have been used in grafting experiments to recapitulate the biology of MPNST in mice. The majority of these experiments studied human MPNST cells in immune-deficient mice. Although some cancer cell types are known to grow only on certain immune-deficient backgrounds (e.g., NSG), MPNST cells can engraft in hosts with residual immune function. MPNST cell lines that have been reported to engraft in mice and the type of mouse background used are listed in Supplemental Table 1. Xenograft models have been primarily used to test candidate therapeutics for MPNST.

### 5.3. Patient-Derived Xenograft (PDX) Models

Culture and xenograft models have been the mainstay of testing novel therapeutics for MPNST over the past 20 years. There is controversy regarding how well these models predict response in patients. This has led to the development of additional models that seek to better emulate the tumor microenvironment. Tumor cells passaged in culture adapt to the lack of extracellular matrix and culture-specific exogenous growth factors. PDX models are created by implanting patient tumor tissue directly into immune-deficient mice, so that the tumor cells grow directly within an in vivo environment. Very few of these models have been published. It is not clear how many have been maintained by passaging for use by other investigators. Bhola et al. [55] isolated tumor tissue from a male young adult NF1 patient and implanted small pieces subcutaneously into male NOD/SCID mice. The explants retained the histological and IHC characteristics of the parental tumor over more than 15 passages [72, 73].

### 5.4. Genetically Engineered Mouse Models (GEMMs)

GEMMs develop MPNST spontaneously, permitting the coevolution of microenvironment and tumor. One GEMM (*Nf1*^−/+^*:Trp53*^−/+^*cis* mice) is being used for preclinical screening of drugs through the NF Therapeutic Consortium (NFTC) [34, 74–76]. The available GEMMs for MPNST use several approaches to initiate tumorigenesis: (1) spontaneous loss of heterozygosity of tumor suppressor genes, (2) expression of oncogenes by nervous system promoters, (3) Cre-lox system for mutation or conditional activation of genes during nerve development, or (4) adenoviral or lentiviral expression of shRNAs (Table 2). Heterozygous mutation of* Nf1* alone is not sufficient to drive MPNST tumorigenesis in mice; however, combining* Nf1* mutation with other mutations (*Trp53*,* Pten*, and* Cdkn2a*) gives rise to MPNST. In addition, MPNST GEMMs have been developed without mutation of* Nf1*, possibly recapitulating sporadic MPNST. The first MPNST GEMM was the* Nf1*^−/+^*:Trp53*^−/+^*cis* mouse [77, 78] with mutated copies of* Nf1* and* Trp53* in* cis* on mouse chromosome 11. Spontaneous loss of the wild-type alleles of these genes initiates tumorigenesis. Combining* Nf1* heterozygosity with loss of* Cdkn2a*, encoding p16^INK4A^ and p19^ARF^, gives rise to MPNST with low penetrance [79]. The Cre-lox system has been used in several GEMMs to mutate* Nf1*,* Trp53*,* Pten*, and/or* Cdkn2a* in cells of the developing nervous system [80–83], as well as to activate mutant Kras [82]. Some GEMMs have combined overexpression of the oncogenes* Egfr* or* Ggfb3* with tumor suppressor mutation by driving oncogene expression in nervous system cells using* CNP* or *P*_0_ promoters, respectively [57, 80, 81, 84, 85]. More recently, MPNSTs have been modeled using injections into adult mouse sciatic nerve. Injection of adenovirus expressing Cre into mice carrying floxed alleles of* Nf1* and* Cdkn2a *drives high-grade MPNST through localized loss of neurofibromin, p16^Ink4a^, and p19^Arf^ in the nerve [86]. Low-grade MPNSTs form with the injection of shRNA for both* Nf1* and* Trp53* into mice that are either mutant for* Nf1* in all Periostin^+^ cells or mutant for* Nf1* in GFAP^+^ cells on a heterozygous mutant* Nf1* background [87]. Injection GEMMs have the advantage that tumorigenesis occurs in a more synchronized and spatially controlled manner; however, they require surgery for every mouse to expose the sciatic nerve for injection. GEMMs show different latencies for MPNST depending on the genes involved* (Cdkn2a, Pten, Trp53,* and* Egfr)* and the method used to mutate genes (shRNA knockdown versus genomic mutation through the Cre-lox system) (Table 2).

## 6. Clinical Trials Advances

Current treatment of MPNST is similar to treatment of soft tissue sarcomas as a whole and relies primarily on local control measures [5]. The only known definitive therapy for MPNST is surgical resection with wide negative margins, which may not be feasible due to variables such as tumor size, location, and/or metastases [7]. The role of adjuvant radiation is not defined; however, it is often recommended for high-grade lesions > 5 cm in size or with marginal excision [8, 88, 89]. For these patients, preoperative radiation should be considered [90]. Although radiation has shown improved local control, no effect on survival has been demonstrated [91, 92]. The role of chemotherapy is not defined. In a prospective study of chemotherapy (ifosfamide, doxorubicin, and etoposide) in NF1 associated and sporadic MPNST, a lower objective response rate was seen in NF1 patients (18%) compared with patients with sporadic MPNST patients (44%), similar to prior studies [93, 94]; however, disease stabilization was achieved in most patients at 4 cycles [95]. The best approach to treatment is by a multidisciplinary team of surgical, medical, and radiation oncologists, radiologists, and pathologists, all with sarcoma expertise. Patients with recurrent, unresectable, or metastatic disease have no known curative options and enrollment in clinical trials should be considered.

The EGFR inhibitor erlotinib was the first targeted agent used in a histology-specific phase II trial for MPNST [96], based on the compelling preclinical observation that EGFR amplification was observed in MPNSTs and that Nf1/p53 murine MPNST were stimulated by EGF and inhibited by EGFR inhibitors [33]. Within 22 months, 24 patients were enrolled, but no activity was demonstrated. Subsequent trials of investigational agents (Table 3) have failed to demonstrate efficacy but show that the outcome for unresectable MPNST is poor with a median progression-free survival of less than 2 months and overall survival of less than 5 months [97–100]. These trials demonstrate that single histology trials in this rare disease are feasible and that MPNST progresses rapidly.

Selection, prioritization, and trial design are key challenges in the clinical development of effective therapies for MPNST. While preclinical drug discovery outpaces clinical development, the time and cost to evaluate promising therapies for MPNST are significant and patient numbers are limited. The Children's Tumor Foundation (CTF) and Neurofibromatosis Therapeutic Acceleration Program (NTAP) sponsor the preclinical NF Therapeutic Consortium (NFTC), which supports the conduct of preclinical trials of targeted therapies in GEMM targeting NF1 manifestations (e.g., MPNST and PN) to prioritize the selection of agents for clinical trials. There are no data yet demonstrating MPNST GEMM as valid surrogates for drug activity in humans. Through clinical consortia initiatives such as the Department of Defense (DoD) NF Clinical Trials Consortium and the SARC (Sarcoma Alliance for Research through Collaboration), therapies identified through these models are being translated into clinical trials specific for MPNST. Cooperative group participation allows for rapid accrual for early phase trials in MPNST. Approaches to accelerating testing of agents guided by preclinical rationale, with efficient endpoints, protocol design, and access to drugs, are needed. In turn, these trials can serve as a means to not only validate the best preclinical models, and information gained in the clinic can be used to help develop new therapeutic approaches at the bench.

## 7. Future Directions

Although the outcome for MPNST has not changed significantly since 2002, the more complete understanding of the natural history of peripheral nerve sheath tumors and of the genomic changes during malignant transformation of PN to ANF and MPNST offers hope for the development of more effective diagnostic, therapeutic, and prevention strategies for MPNST. Whole-body MRI and PET imaging may have utility for risk stratification and for implementation of surveillance and medical/surgical interventions as potential preventative therapies and for monitoring treatment response in large, irregular-shaped tumors. Research priorities should focus on the role of whole-body MRI to screen for PN-related tumor load and on longitudinal imaging to detect lesions concerning malignant transformation, such as DNL. The natural history of ANF needs to be better understood, including its clinical presentation, incidence of malignant transformation of DNL to ANF, and the role for timing and extent (wide versus limited) of surgical excision of these transitional tumors, while resource-intensive, prospective, longitudinal studies of individuals with NF1 and PN with whole-body MRI and other imaging modalities coupled with genomic and immunohistological data and collection of blood samples for potential biomarker development are predicted to have great value in advancing approaches to the diagnosis, treatment, and prevention of MPNST.

Great strides have been made in the development of preclinical models for understanding disease pathogenesis and drug testing in MPNST. Translating and validating preclinical models will require developing validated biomarkers of disease and outcome measures using new technologies that can be incorporated into clinical trials. The search for MPNST biomarkers must have new urgency. Circulating tumor DNA is essentially unstudied in MPNST and may offer great promise to screen and detect early cancers, score treatment response, and identify tumor recurrence. The importance of epigenetic mechanisms in MPNST pathogenesis has been underappreciated until the advent of comprehensive genomic studies which have offered clues to future therapies. An international MPNST database with phenotypic, genotypic, and treatment data is needed to share findings and inform next steps for research efforts and treatment strategies [104]. The tumor phenotype data should be comprehensive and include complete characterization of the tumors from clinical pathologists with expertise in sarcoma. To that end, standard and broadly accepted definitions of what constitutes benign cellular neurofibroma, DNL, ANF, low-grade MPNST, and high-grade MPNST need to be established. Molecular data needed for the MPNST database include constitutional DNA, tumor DNA, tumor expression patterns, circulating cell-free DNA, and possibly metabolic activity of the tumor. Treatment and outcome data need to be collated with the genotype-phenotype information in the database. Innovative clinical trial designs with efficient endpoints to accelerate testing of new drugs and access to novel agents for testing in combination are also needed.

## Supplementary Material

NF1 or sporadic MPNST cell lines from primary or metastatic human and mice tumors have been described in the literature to varying degrees and are listed in Supplemental Table 1.

## Figures and Tables

**Table 1:** Summary of progress in preclinical, clinical, and therapeutic MPNST research and clinical management since the 2002 international consensus conference.

Characteristic	2002	2016
Natural history of PN growth	(i) Unknown (ii) May be erratic	(i) Well characterized (ii) Identification of distinct nodular lesions (DNL) with different growth pattern

Imaging	(i) Role of FDG-PET unclear (ii) FLT PET should be considered	(i) FDG-PET has clear role (ii) FLT-PET under evaluation

Pathology	(i) ANF do not fit in category (ii) Locally aggressive (iii) Do not metastasize	(i) Identification of ANF as MPNST precursor

Risk for transformation ↑	(i) Nodular PN, large central PN, NF neuropathy	(i) Distinct nodular, FDG-avid lesions

Pathogenesis	(i) *NF1* microdeletion (ii) *p27, p53, p16*	(i) *CDKN2A/B* (ii) *SUZ12*, *EED*

Mouse models	(i) Briefly mentioned	(i) Preclinical trials consortium using GEMM

Chemotherapy targeted therapy	(i) Very few, if any, MPNST-specific data	(i) Prospective trial of chemotherapy completed (ii) MPNST-specific targeted trials ongoing (iii) SARC and NF clinical trials consortium

Access to tissue	(i) Importance of tissue banking	(i) CTF NF biobank

Data collection	(i) International database recommended	(i) No international database established

ANF: atypical neurofibroma; DNL: distinct nodular lesion; FDG-PET: fluorodeoxyglucose positron emission tomography; FLT-PET: fluorothymidine positron emission tomography; GEMM: genetically engineered mouse model; PN: plexiform neurofibroma; SARC: Sarcoma Alliance for Research through Collaboration (research and advocacy group); CTF: Children's Tumor Foundation.

**Table 2:** Genetically engineered mouse models (GEMMs).

Tumor suppressors mutated	Method of mutation	Oncogenes overexpressed	Promoter overexpressed	Grade	Latency (months)	Penetrance (%)	REF
Nf1^−/+^; Ink4a^−/−^; Arf^−/−^	Germline^1^			High	6.5	26	[79]
Nf1^flox/flox^; Pten^flox/flox^	Cre (Dhh^+^ cells)			High	0.5	92	[80]
Nf1^flox/flox^; Pten^flox/+^	Cre (Dhh^+^ cells)			Low	5.7	42	[80]
Nf1^flox/+^; Pten^flox/flox^	Cre (Dhh^+^ cells)			Low	5.8	82	[80]
Nf1^flox/flox^ + Nf1; p53shRNA	Cre (Periostin^+^ cells)^4^			Low	6.1	56	[87]
Nf1^flox/−^ + Nf1; p53shRNA	Cre (GFAP^+^ cells)^4^			Low	3.0	73	[87]
Nf1^flox/flox^; Ink4a^flox/flox^; Arf^flox/flox^	Cre (injection)			High	4.1	100	[86]
Nf1^−/+^:Trp53^−/+^	Germline^2^			High	5	81	[77, 78]
Nf1^flox/flox^	Cre (Dhh^+^ cells)	EGFR	CNP	High	~6	33	[81]

Trp53^−/+^	Germline^3^	EGFR	CNP	Low-high	9.5	19	[84]
Pten^flox/+^	Cre (GFAP^+^ cells)	Kras-G12D	lox-STOP-lox^5^	High	~6	100	[82]
Pten^flox/flox^	Cre (Dhh^+^ cells)	EGFR	CNP	High	<1	100	[83]
Trp53^−/+^	Germline^3^	GGF*β*3	P0	Low-high	7.5	95	[57]
NA	NA	GGF*β*3	P0	ND	8.7	71	[85]

^1^Spontaneous loss of NF1; ^2^spontaneous loss of NF1 and p53; ^3^spontaneous loss of p53; ^4^injection of shRNA into sciatic nerve; ^5^activation by Cre (GFAP^+^ cells).

NA: not applicable; ND: not determined.

**Table 3:** Targeted agents for treatment of MPNST: previous and ongoing clinical trials.

Drug	Target	Phase	*n*	Population	Outcome	Results	Ref.
Erlotinib	EGFR	II	24	≥18 y Refractory	Response WHO [101]	19/20 pts. PD at 2 months 1 SD	[96]
Sorafenib	C-Raf B-Raf VEGFR2 C-Kit PDGFR	II	12	≥18 y Refractory	Response RECIST [102]	No responses; median PFS 1.7 months	[97]
Imatinib	C-Kit PDGFR VEGFR	II	7	>10 y Refractory	Response RECIST [102]	No responses; 1 SD	[98]
Dasatinib	C-Kit SRC	II	14	≥13 y Refractory	Response CHOI [103]	No response or SD	[99]
Alisertib	AURKA	II	10	≥18 y Refractory	Response RECIST [102]	No response PFS 13 weeks	[100]
Bevacizumab/RAD001	Angiogenesis/mTOR	II	—	≥18 y Refractory	Response WHO [101]	Currently ongoing	—
Ganetespib/Sirolimus	Hsp90 mTOR	I/II	—	≥16 y Refractory	Response WHO [101]	Currently ongoing	—
