# Supplementary material for: Malignant Peripheral Nerve Sheath Tumors State of the Science: Leveraging Clinical and Biological Insights into Effective Therapies
Source: Sarcoma. 2017 May 16;2017:7429697. doi: 10.1155/2017/7429697 (PMC5448069; doi:10.1155/2017/7429697)
Supplement: Supplementary file 1 — NF1 or sporadic MPNST cell lines from primary or metastatic human and mice tumors have been described in the literature to varying degrees and are listed in Supplemental Table 1. [file 7429697.f1.docx]

**Supplemental Table 1. Malignant Peripheral Nerve Sheath Tumor Cell Lines and Xenograft/Orthograft Models**

| **Cell Line** | **NF1 or Spor** | **Prim or Met** | **Sex** | **SCID** | **Nude** | **NIH III** | **NSG** | **NOD/SCID** | **s.c.** | **i.n.** | **t.v.** | **i.p.** | **REF** |
| --- | --- | --- | --- | --- | --- | --- | --- | --- | --- | --- | --- | --- | --- |
| **Human** |  |  |  |  |  |  |  |  |  |  |  |  |  |
| 88-14 | NF |  |  |  |  |  |  |  |  |  |  |  | [1] |
| 88-3 | NF |  |  |  |  |  |  |  |  |  |  |  | [1] |
| 90-8 | NF |  |  |  |  |  |  |  |  |  |  |  | [1] |
| FMS-1 | NF | Prim | F | X | X |  |  | X | X |  |  |  | [2] |
| FUSFT8611 | Spor | Met | M | X | X |  |  |  | X |  |  |  | [3] |
| FUSFT8710 | NF | Prim | F |  |  |  |  |  |  |  |  |  | [3] |
| FUSFT9817 | Spor | Prim | F | X | X |  |  |  | X |  |  |  | [3] |
| Hs-Sch-2 | Spor |  | F |  | X |  |  | X | X |  |  |  | [4] |
| MPNST642 |  |  |  | X |  |  |  |  | X |  |  |  | [5] |
| MPNST724 |  |  |  | X |  |  |  |  | X |  |  |  | [6] |
| NMS-2 | NF | Prim | M |  | X |  |  |  | X |  |  |  | [7] |
| NMS-2PC | NF | Met | M |  | X |  |  | X | X |  |  |  | [7] |
| S462 | NF |  | F |  | X |  |  |  | X | X |  |  | [8] |
| S462.TY | NF |  | F |  | X |  |  |  | X |  |  |  | [9] |
| S520 |  |  |  |  |  |  |  |  |  |  |  |  | [8] |
| S805 |  |  |  |  |  |  |  |  |  |  |  |  | [8, 10] |
| sNF02.2 | NF | Met | M |  |  |  |  |  |  |  |  |  | [11] |
| sNF94.3 | NF | Met | F | X |  |  |  |  |  | X |  |  | [12] |
| sNF96.2 | NF | Prim | M | X |  |  |  |  |  | X |  |  | [13] |
| ST88-14 | NF |  | M | X | X | X | X |  | X | X |  |  | [14] |
| STS26T | Spor | Met | F | X | X | X | X |  | X | X | X | X | [9, 15] |
| T265 | NF |  |  | X | X | X | X |  |  | X |  |  | [16] |
| YST-1 |  |  | F |  | X |  |  |  | X |  |  |  | [17] |
| **Mouse** |  |  |  |  |  |  |  |  |  |  |  |  |  |
| 32-5-30 | NPcis | Prim |  |  |  |  |  |  |  |  |  |  | [18] |
| 35-1-2 | NPcis | Prim |  | X |  |  |  |  | X |  |  |  | [10] |
| 37-18-4 | NPcis | Prim |  |  |  |  |  |  |  |  |  |  | [19] |
| 37-3-18 | NPcis | Prim |  |  |  |  |  |  |  |  |  |  | [10] |
| 38-24-12 | NPcis | Prim |  |  |  |  |  |  |  |  |  |  | [10] |
| 39-2-11 | NPcis | Prim |  |  |  |  |  |  |  |  |  |  | [18] |
| 6IE4 | NPcis | Prim |  |  |  |  |  |  |  |  |  |  | [10] |
| JW3 | NPcis | Prim | F |  |  |  |  |  |  |  |  |  | [20] |
| JW6 | NPcis | Prim | F |  |  |  |  |  |  |  |  |  | [20] |
| MPNST6IEPVI | NPcis | Prim |  | X |  |  |  |  | X |  |  |  | [21] |

**Abbreviations:** NF1, Neurofibromatosis Type 1; Spor, Sporadic; Prim, Primary; Met, Metastatic; M, male; F, female; SCID, severe combined immunodeficiency mice; NOD, non-obese diabetic mice; NSG, NOD/SCID/Il2r-gamma mice; NIH III, mutant for nude, xid, and bg; s.c., subcutaneous; i.n., intraneural; t.v., tail vein; i.p., intraperitoneal; REF, Reference.

REFERENCES:

1. DeClue, J.E., et al., *Abnormal regulation of mammalian p21ras contributes to malignant tumor growth in von Recklinghausen (type 1) neurofibromatosis.* Cell, 1992. 69(2): p. 265-73.

2. Hakozaki, M., et al., *Establishment and characterization of a novel human malignant peripheral nerve sheath tumor cell line, FMS-1, that overexpresses epidermal growth factor receptor and cyclooxygenase-2.* Virchows Arch, 2009. 455(6): p. 517-26.

3. Aoki, M., et al., *Establishment of three malignant peripheral nerve sheath tumor cell lines, FU-SFT8611, 8710 and 9817: conventional and molecular cytogenetic characterization.* Int J Oncol, 2006. 29(6): p. 1421-8.

4. Sonobe, H., et al., *A new human malignant peripheral nerve sheath tumour-cell line, HS-sch-2, harbouring p53 point mutation.* Int J Oncol, 2000. 17(2): p. 347-52.

5. Ghadimi, M.P., et al., *Survivin is a viable target for the treatment of malignant peripheral nerve sheath tumors.* Clin Cancer Res, 2012. 18(9): p. 2545-57.

6. Torres, K.E., et al., *Activated MET is a molecular prognosticator and potential therapeutic target for malignant peripheral nerve sheath tumors.* Clin Cancer Res, 2011. 17(12): p. 3943-55.

7. Imaizumi, S., et al., *Characterization and chemosensitivity of two human malignant peripheral nerve sheath tumour cell lines derived from a patient with neurofibromatosis type 1.* Virchows Arch, 1998. 433(5): p. 435-41.

8. Frahm, S., et al., *Sulindac derivatives inhibit cell growth and induce apoptosis in primary cells from malignant peripheral nerve sheath tumors of NF1-patients.* Cancer Cell Int, 2004. 4(1): p. 4.

9. Mahller, Y.Y., et al., *Tissue inhibitor of metalloproteinase-3 via oncolytic herpesvirus inhibits tumor growth and vascular progenitors.* Cancer Res, 2008. 68(4): p. 1170-9.

10. Bodempudi, V., et al., *Ral overactivation in malignant peripheral nerve sheath tumors.* Mol Cell Biol, 2009. 29(14): p. 3964-74.

11. Li, Y., et al., *Notch and Schwann cell transformation.* Oncogene, 2004. 23(5): p. 1146-52.

12. Perrin, G.Q., et al., *Plexiform-like neurofibromas develop in the mouse by intraneural xenograft of an NF1 tumor-derived Schwann cell line.* J Neurosci Res, 2007. 85(6): p. 1347-57.

13. Perrin, G.Q., et al., *An orthotopic xenograft model of intraneural NF1 MPNST suggests a potential association between steroid hormones and tumor cell proliferation.* Lab Invest, 2007. 87(11): p. 1092-102.

14. Fletcher, J.A., et al., *Diagnostic relevance of clonal cytogenetic aberrations in malignant soft-tissue tumors.* N Engl J Med, 1991. 324(7): p. 436-42.

15. Dahlberg, W.K., et al., *Radiosensitivity in vitro of human soft tissue sarcoma cell lines and skin fibroblasts derived from the same patients.* Int J Radiat Biol, 1993. 63(2): p. 191-8.

16. Muja, N., et al., *Identification and functional characterization of thromboxane A2 receptors in Schwann cells.* J Neurochem, 2001. 78(3): p. 446-56.

17. Nagashima, Y., et al., *Establishment of an epithelioid malignant schwannoma cell line (YST-1).* Virchows Arch B Cell Pathol Incl Mol Pathol, 1990. 59(5): p. 321-7.

18. Mashour, G.A., et al., *Differential modulation of malignant peripheral nerve sheath tumor growth by omega-3 and omega-6 fatty acids.* Oncogene, 2005. 24(14): p. 2367-74.

19. Liu, T.C., et al., *Dominant-negative fibroblast growth factor receptor expression enhances antitumoral potency of oncolytic herpes simplex virus in neural tumors.* Clin Cancer Res, 2006. 12(22): p. 6791-9.

20. Turbyville, T.J., et al., *Schweinfurthin A selectively inhibits proliferation and Rho signaling in glioma and neurofibromatosis type 1 tumor cells in a NF1-GRD-dependent manner.* Mol Cancer Ther, 2010. 9(5): p. 1234-43.

21. Lopez, G., et al., *HDAC8, A Potential Therapeutic Target for the Treatment of Malignant Peripheral Nerve Sheath Tumors (MPNST).* PLoS One, 2015. 10(7): p. e0133302.
